# Social vulnerability and outcomes of head and neck cutaneous squamous cell carcinoma: A retrospective cohort study

**DOI:** 10.1016/j.jdin.2024.07.025

**Published:** 2024-08-31

**Authors:** Lillian McCampbell, David Jun Fei-Zhang, Daniel Chelius, Ling-Lun Bob Hsia, Jeffrey Rastatter, Anthony Sheyn

**Affiliations:** a Department of Otolaryngology-Head and Neck Surgery, University of Tennessee Health Science Center, Memphis, Tennessee; b Northwestern University Feinberg School of Medicine, Chicago, Illinois; c Department of Otolaryngology-Head and Neck Surgery, Pediatric Thyroid Tumor Program and Pediatric Head and Neck Tumor Program, Baylor College of Medicine, Texas Children's Hospital, Houston, Texas; d Department of Dermatology, Medical University of South Carolina, Charleston, South Carolina; e Division of Pediatric Otolaryngology, Ann & Robert H. Lurie Children's Hospital of Chicago, Chicago, Illinois; f Department of Pediatric Otolaryngology, Le Bonheur Children's Hospital, Memphis, Tennessee; g Department of Pediatric Otolaryngology, St. Jude Children's Research Hospital, Memphis, Tennessee

**Keywords:** cutaneous squamous cell carcinoma, head and neck, social determinants of health

*To the Editor:* Cutaneous squamous cell carcinoma (cSCC) is the second most prevalent cutaneous malignancy and most common cause of death from nonmelanoma skin cancers, especially of the head-neck region.^1^ Beyond notable clinical factors, social determinants of health (SDoH) have seen associations with poor cSCC outcomes, primarily focused on socioeconomic status and race/ethnicity.^2^ However, the summative impact of SDoH beyond these singular factors has been seldom investigated. Using the Centers for Disease Control and Prevention-Social Vulnerability Index (SVI), we assessed the relationships of the sum of SDoH vulnerabilities more broadly and head-neck cSCC disparities in the United States.

The SVI comprises composite, county-level SDoH-metrics (ie, total SDoH-vulnerability) of 15 factors across socioeconomic status, minority language, household composition, and housing-transportation themes by vulnerability-ranked scores across the United States while adjusting for sociodemographic variation (ie, weighted averages dynamically changed based on geography and adjusted for in total SDoH-vulnerability). After linking these with county-of-residence of cSCC patients in the Surveillance-Epidemiology-End Results database, univariate linear and logistic regressions of this composite SVI metric were used to assess survival, follow-up/surveillance, treatment, late-staging, and high-grading. Univariate approaches (over multivariate) allowed preservation of the composite SVI-scores’ dynamic weights while retaining a “multivariate” interpretation through its joint consideration of 15-SDoH factors. Community/county-level confounders were accounted for through this dynamic weighting across US sociodemographic contexts. Respectively, relative mean-% and odds ratios described the clinical significances of analyses.

For 16,865 adults with head-neck cSCC from 1975-2017, increasing from the lowest to highest relative quintiles of total SDoH-vulnerability conferred survival decreases of 25.82% (113.27 months to 84.02 months; *P* < .001 by regression) and follow-up/surveillance decreases of 21.84% (118.97 months to 93.00 months; *P* < .001 by regression) (Figs 1 and 2). Specific SDoH-vulnerabilities in socioeconomic status, minority language, household composition, and housing-transportation showed different levels of contribution to total SDoH-vulnerability (Figs 1 and 2). With increasing total SDoH vulnerability, increased odds of late staging (Odds ratio 1.17; 95% CI 1.07-1.28), high-grading (1.09; 1.03-1.14), and radiation treatment (1.13; 95% CI 1.08-1.17) but not surgery were observed.

**Fig 1:**
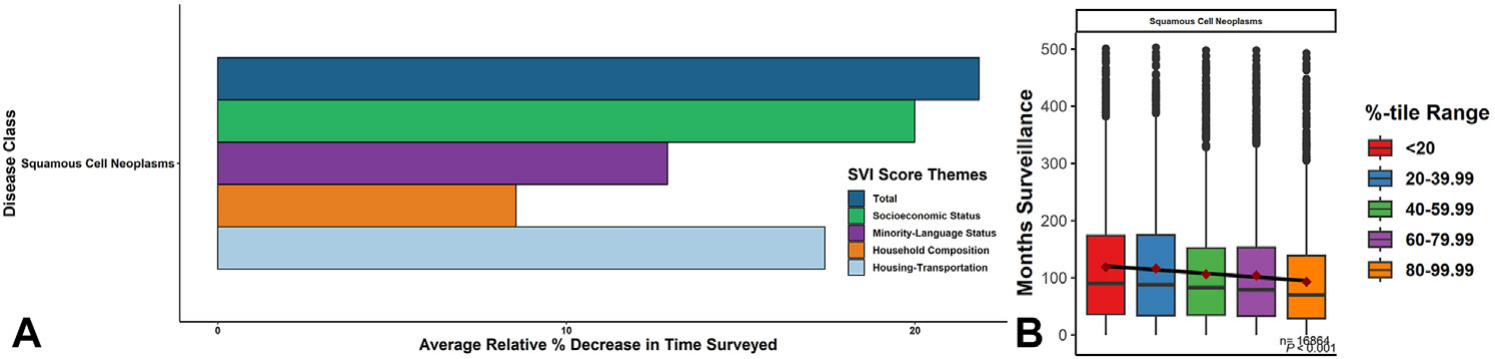
Relative decreases in follow-up surveillance period with increasing social vulnerability. **A,** Mean follow-up surveillance period in months was calculated for each vulnerability quintile within total-SVI and each of its subcomponent SDoH-theme categories. The difference between the lowest vulnerability and highest vulnerability quintiles was then calculated and converted into relative percentages. **B,** Significance of these differences was assessed via linear regression across increasing relative social vulnerability quintiles. *SDoH*, Social determinants of health; *SVI*, Social Vulnerability Index.

**Fig 2:**
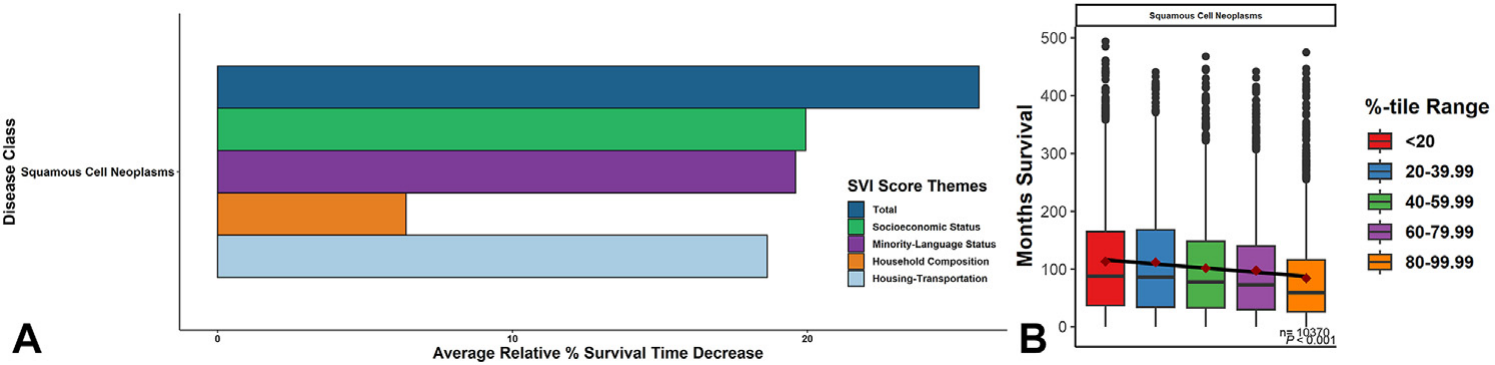
Relative decreases in survival period with increasing social vulnerability. **A,** Mean survival period in months was calculated for each vulnerability quintile within total-SVI and each of its subcomponent SDoH-theme categories. The difference between the lowest vulnerability and highest vulnerability quintiles was then calculated and converted into relative percentages. **B,** Significance of these differences was assessed via linear regression across increasing relative social vulnerability quintiles. *SDoH*, Social determinants of health; *SVI*, Social Vulnerability Index.

This study principally highlights the utility of the SVI to measure broader, summative SDoH-vulnerability associations of a patient’s community with head-neck cSCC disparities. This expands prior SDoH-understandings by contextualizing and adjusting for various community-level confounding SDoH-effects while highlighting specific mechanisms of care and prognostic disparities that are influenced by total SDoH-vulnerabilities.

Unlike prior observations focusing on race/ethnicity for its effects on discordant rates of cSCC metastasis,^2^^,^^3^ our results showcase that patients with higher total SDoH-vulnerability share increased rates of late-staging and high-grading on diagnosis beyond aspects of minoritized race/ethnicity. Similarly, past investigations also singularly focused on socioeconomic status but only observed significant effects among elderly males for survival.^4^ Our investigation provides more robust considerations of varied SDoH-factors to showcase composite SDoH-vulnerability influence while adjusting for varied community settings. But, our study must consider its limitations of using county-level metrics to assess total SDoH-vulnerability due to data restrictions of more-specific geolinkage variables.

In sum, this SVI-based, retrospective study showcased how total county-level SDoH-vulnerability observed head-neck cSCC care and prognostic disparities across wider swathes of cSCC patients than prior observations. As cSCC disparities are shared more broadly, public health initiatives should more strongly address this universal need.^5^

## Conflicts of interest

Dr Chelius reported receiving a coordinator stipend from the American Academy of Otolaryngology outside the submitted work. Drs McCampbell, Fei-Zhang, Bob Hsia, Rastatter, and Sheyn have no conflicts of interest to declare.
